# Influence of Sulfate-Reducing Bacteria on the Corrosion Residual Strength of an AZ91D Magnesium Alloy

**DOI:** 10.3390/ma7107118

**Published:** 2014-10-21

**Authors:** Xianyong Zhu, Yaohui Liu, Qiang Wang, Jiaan Liu

**Affiliations:** 1 College of Mechanical Science and Engineering, Jilin University, Changchun 130025, Jilin, China; 2 Key Laboratory of Automobile Materials (Jilin University), Ministry of Education and College of Materials Science and Engineering, Jilin University, Changchun 130025, Jilin, China; E-Mails: liuyh@jlu.edu.cn (Y.L.); wangqiang@jlu.edu.cn (Q.W.); liuja@jlu.edu.cn (J.L.)

**Keywords:** corrosion residual strength, magnesium alloy, microbiologically influenced corrosion, pitting corrosion

## Abstract

In this paper, the corrosion residual strength of the AZ91D magnesium alloy in the presence of sulfate-reducing bacteria is studied. In the experiments, the chemical composition of corrosion film was analyzed by a scanning electron microscope with energy dispersive X-ray spectroscopy. In addition, a series of instruments, such as scanning electronic microscope, pH-meter and an AG-10TA materials test machine, were applied to test and record the morphology of the corrosion product, fracture texture and mechanical properties of the AZ91D magnesium alloy. The experiments show that the sulfate-reducing bacteria (SRB) play an important role in the corrosion process of the AZ91D magnesium alloy. Pitting corrosion was enhanced by sulfate-reducing bacteria. Corrosion pits are important defects that could lead to a significant stress concentration in the tensile process. As a result, sulfate-reducing bacteria influence the corrosion residual strength of the AZ91D magnesium alloy by accelerating pitting corrosion.

## 1. Introduction

Magnesium alloys are widely used in the automotive industry and civil industry to replace conventional metallic materials, which is attributed to their excellent castability and machinability [1,2,3,4,5,6]. AZ91D magnesium alloy is representative of commercial magnesium alloys [7,8,9]. In recent years, bad corrosion resistance still restricts the application of AZ91D magnesium alloy [10,11,12,13,14]. Although a great deal of work has been done to reveal the corrosion mechanisms of this magnesium alloy and to try to find better approaches to solve the bad corrosion problem, the corrosion problem is still ahead of us.

Pitting corrosion is an objectionable corrosion type, which can easily cause the failure of magnesium alloy. As is well known, corrosion pits are important volume-scale defects, which could result in a significant stress concentration when the structure’s components are in service. Sometimes, serious stress concentration is fatal to the structure components. In previous studies [15,16,17], it was found that pitting corrosion has a remarkable influence on the mechanical properties of magnesium alloy. In some situations, this pitting corrosion might last for a long time until the structure components fail. Aggressive environments may be everywhere, and therefore, corrosion is inevitable. Among the environmental factors, bacterial metabolism is an important factor that influences the corrosion behavior of magnesium alloy.

Sulfate-reducing bacteria (SRB) are common anaerobic bacteria, which can live in soil, water and damp air. They can use an organic carbon source, a nitrogen source and inorganic sulfates for their metabolites. Generally, SRB could change the corrosion medium through their metabolic process. In recent years, the microbiologically influenced corrosion caused by SRB was reported widely [18,19]. In contrast to human beings, SRB have a strong vitality, and therefore, they can induce some destruction if they are ignored. The corrosion residual strength, which is defined as the tensile strength of the alloy tested after corrosion, is an important parameter to characterize the corrosion resistance of the magnesium alloy [17]. However, there has been little research on the corrosion residual strength of AZ91D magnesium alloy in the presence of sulfate-reducing bacteria to date. Therefore, in this paper, we focus on the mechanism and the behavior for the change of the corrosion residual strength of the AZ91D magnesium alloy in the presence of sulfate-reducing bacteria.

## 2. Results and Discussion

### 2.1. Morphology of the Corrosion Product

**Figure 1:**
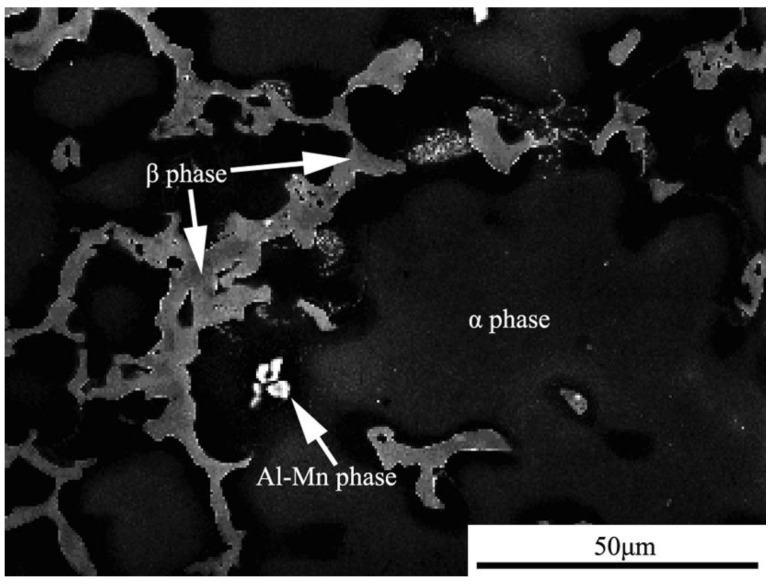
Microstructure of the AZ91D magnesium alloy.

**Figure 2:**
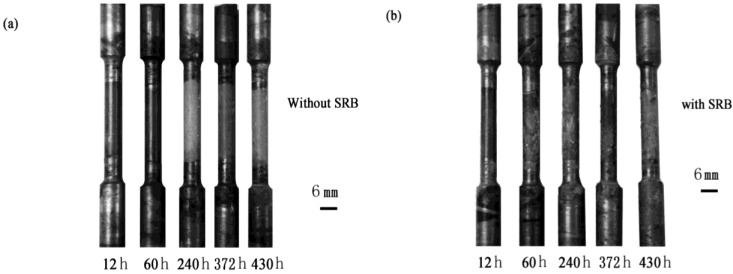
Morphology of the corrosion product of the test bars (**a**) in sterile corrosion medium; and (**b**) in sulfate-reducing bacteria (SRB) corrosion medium.

AZ91D magnesium alloy is a dual-phase alloy, which consists of the α phase (Mg-Al solid solution) and β phase (intermetallic compound), as shown in Figure 1. In the alloy matrix, the β phase distributes around the α phase. Figure 2 shows the development of the morphology of the corrosion product. In the SRB corrosion medium, the corrosion pits nucleated and grew quickly. By contrast, the corrosion pits nucleated and grew slowly in the sterilized corrosion medium. A series of images of the samples were captured in different corrosion stages. In the first 12 h, there are almost no corrosion pits that can be found on the sample surface (Figure 2). After 60 h of immersion, a number of corrosion pits appeared on the surface of the samples that were immersed in the SRB corrosion medium. However, corrosion pits also cannot be found on the sample surface immersed in sterilized corrosion medium. After exposure for 240 h, several corrosion pits appeared on the sample surface, as shown in Figure 2a. Meanwhile, the sample immersed in the SRB corrosion medium was corroded seriously, and many corrosion pits appeared due to rapid corrosion. In general, the variety of the morphology of the corrosion product indicated that SRB is an important factor influencing the corrosion of magnesium alloy.

### 2.2. Corrosion Residual Strength

**Figure 3:**
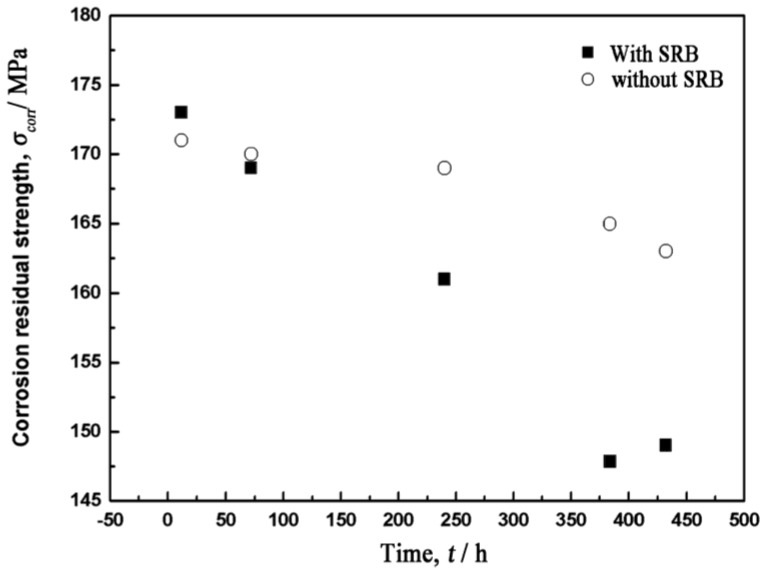
The corrosion residual strength of the AZ91D magnesium alloy in the presence of SRB drops faster than in the absence of SRB.

**Figure 4:**
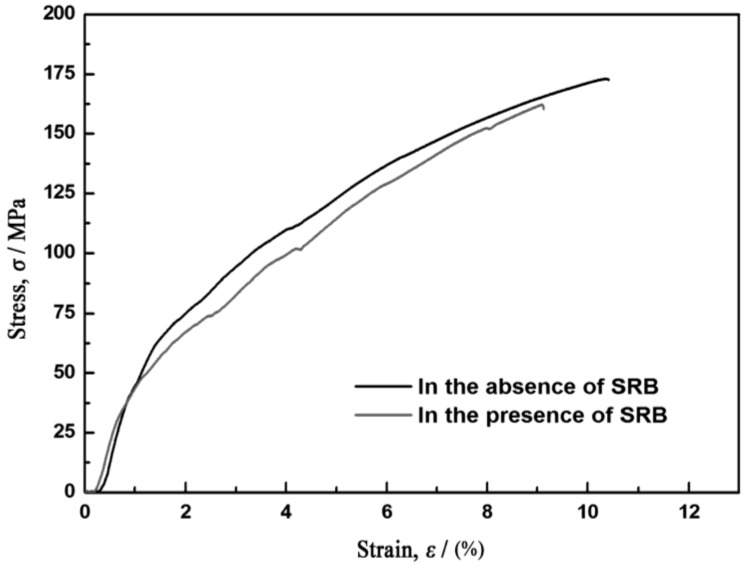
True stress-strain curve of the AZ91D magnesium alloy after 240 h of immersion in the corrosion medium in the presence of SRB and in the absence of SRB.

Figure 3 shows the variation of the corrosion residual strength with time. It is worth pointing out that the corrosion residual strength of the AZ91D magnesium alloy immersed in the SRB corrosion medium drops faster than in the sterilized corrosion medium. After 240 h of immersion, the corrosion residual strength of the AZ91D magnesium alloy drops remarkably. Thus, the stress-strain curve of the AZ91D magnesium alloy after 240 h of immersion is given in Figure 4 for the purpose of clarifying the effect of SRB on the tensile behavior of the magnesium alloy. It is significant to make a comparison between the two corrosion environments. In the presence of SRB, both the fracture stress and the strain drop simultaneously according to the curves in Figure 4. It can be deduced that the SRB played an important role in the variation of the corrosion residual strength.

**Figure 5:**
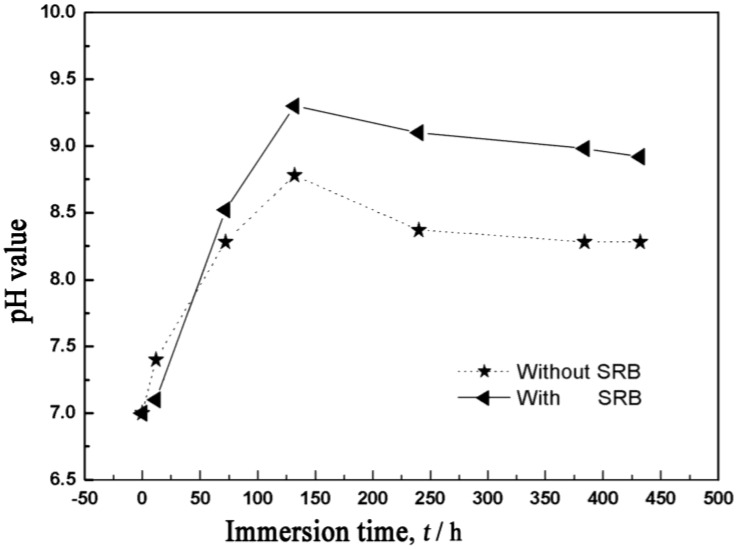
Variation of the pH value with time.

The pH value is also an important factor that influences the corrosion behavior of the magnesium alloy. In Figure 5, the pH value of the SRB corrosion medium rises faster than the sterilized corrosion medium. As is well known, hydrogen evolution is the major cathodic reaction in the corrosion process of the magnesium alloy, and therefore, raising the pH value contributes to the stabilization of the corrosion film. However, actually, it was not. In the SRB corrosion medium, the pH value is higher than the sterilized corrosion medium. The variation of the pH value can be attributed to the metabolic process of SRB. In the SRB corrosion medium, the reaction can be given as follows:


H_2_O → H^+^ + OH^−^ (1)

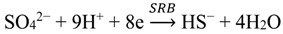
(2)


HS^−^ + H^+^ → H_2_S↑ (3)

In the metabolic process, H^+^ is consumed in the above reaction and the pH value of the corrosion medium rises with time. However, pitting corrosion still occurred in the experiment. Maybe another reason is the cause of the pitting corrosion of the AZ91D magnesium alloy.

It is noted that the reduction of sulfate by SRB is coupled to an oxidation reaction, for instance that of organic matter, e.g., lactate ions. One of the corresponding reactions is: 


2CH_3_CHOHCOO^−^ + 4H_2_O → 2CH_3_COO^−^ + 2HCO_3_^−^ + 10H^+^+ 8e^−^ (4)

It can be seen that this organic reaction produces 10H*^+^*. Therefore, the metabolic activity of SRB is responsible for the increase of pH.

### 2.3. Fractography Analysis

**Figure 6:**
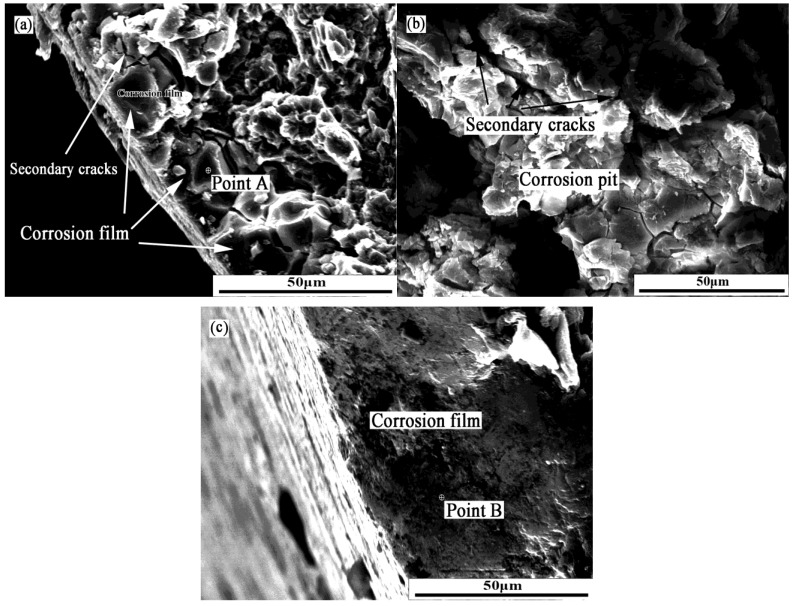
Fractography of test samples: (**a**) tensile fracture of the samples immersed in the sterile corrosion medium; (**b**) tensile fracture of the samples immersed in the SRB corrosion medium; and (**c**) morphology of the tensile fracture with corrosion film in the presence of SRB.

Figure 6 shows the fracture morphology of the samples immersed in the corrosion medium for 240 h. In Figure 6a, many secondary cracks emerge on the fracture surface distributed near the interface between the corrosion film and the alloy matrix. The corrosion film covers the matrix and protects the matrix against further corrosion. Actually, the deformability of the corrosion film is different from the alloy matrix in the tensile process. Different deformability facilitates the formation of the secondary cracks at the interface. Therefore, the interface between the corrosion film and the alloy matrix was the crack source in the tensile process. Finally, the growth of the microcracks caused the failure of the sample.

Figure 6b shows a corrosion pit on the tensile fracture surface, and part of the fracture surface was covered by the corrosion products. The width of the secondary cracks is obviously bigger than that in Figure 6a. It can be inferred that there is a higher stress concentration at this position in the tensile process. Many secondary cracks perpendicular to the main crack appeared at the corrosion pit. This result indicated that the corrosion pit was the crack source in the tensile process. Usually, pitting corrosion easily results in the change of the sample surface and leaves a volume-scale defect, which is fatal to the AZ91D magnesium alloy. This kind of defect can accelerate the failure of structure components. Thus, the microbiologically-induced corrosion directly influences the corrosion residual strength of magnesium alloy. Figure 6c shows the morphology of the tensile fracture with corrosion film in the presence of SRB. It is clear that the corrosion film covers the whole surface of the alloy. The corrosion film is important for the corrosion resistance of the magnesium alloy, because some special chemicals can help magnesium alloy to form a passive film, and they are beneficial to corrosion resistance. To explore the effect of SRB on the corrosion process of the alloy, the composition of the corrosion film is also studied in the next experiment.

### 2.4. Composition of the Corrosion Film

Many factors influence the corrosion behavior of the AZ91D magnesium alloy, such as corrosion medium, corrosion film, alloy composition, and so forth. Among these factors, corrosion film is important, and it always depends on its composition. Some special chemicals can help the magnesium alloy to form a passive film, for example, PO_4_^3^^−^. In corrosive environments, the passive film can protect the alloy matrix against corrosion. However, a bad corrosion film cannot act as a good protector. It may lead to further corrosion. Here, a scanning electronic microscope with the X-ray energy dispersive spectrum was adopted to analyze the composition of the corrosion film in order to reveal the role of SRB in the corrosion process.

**Figure 7:**
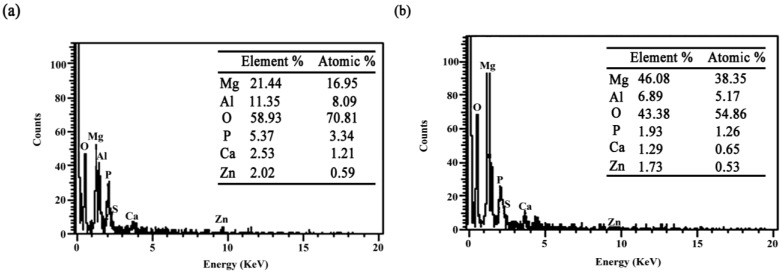
Compositions of the corrosion film formed (**a**) in the absence of SRB in Figure 6a at Point A; and (**b**) in the presence of SRB in Figure 6c at Point B.

Figure 7a shows the composition of the corrosion film at Point A in Figure 6a, and simultaneously, the composition of the corrosion film at Point B in Figure 6c is given in Figure 7b. It can be found that the content of element P at Point A is obviously higher than that at Point B. In addition, the state of the corrosion film at Point A is apparently different from that at Point B. The decrease of element P in the corrosion film may be an important reason for the pitting corrosion of the AZ91D magnesium alloy in the SRB corrosion medium. Undoubtedly, the metabolic process of SRB should be responsible for the variation of element P in the corrosion film. As a result, it was SRB that accelerated the drop in the corrosion residual strength. Some papers focused on the effect of the metabolism of sulfate on the corrosion behavior of magnesium alloy. Actually, it is difficult to clarify the effect of sulfate metabolism on the corrosion of magnesium alloy in biologically-influenced corrosion.

Figure 6a,b provide some useful information related to phosphate metabolism. Phosphate is one of the most important metabolic substances for bacteria. It is the main component of the biological membrane. Therefore, an amount of phosphate was consumed in the SRB corrosion medium. The variation of the corrosion medium directly influenced the composition of the corrosion film. As a result, SRB play an important role in the corrosion process of the AZ91D magnesium alloy. Additionally, the influence is inevitable in the presence of SRB.

### 2.5. Mechanism Discussion

The corrosion behavior of the metal usually depends on the base metal, the corrosion film and the corrosion medium. Once the base metal and the corrosion medium are determined, the corrosion film becomes the main factor that influences the corrosion process of the metal. In this paper, the AZ91D magnesium alloy was immersed in the corrosion medium during the experiment. The corrosion film significantly affected the corrosion process of the AZ91D magnesium alloy. In the SRB corrosion medium, the samples were susceptible to pitting corrosion. However, in the sterilized corrosion medium, the samples have better corrosion resistance. According to Figure 7a,b, phosphate is a vital component of the corrosion film. Moreover, phosphate is also an important metabolic substance for SRB in the culture medium. In the previous study, Fang* et al.* [20] proved that NH_4_MgPO_4_·6H_2_O is the main corrosion product in the SRB corrosion medium. The corrosion product can be given in the following reaction:


Mg^2+^ + PO_4_^3−^ + NH_4_^+^ + 6H_2_O → NH_4_MgPO_4_·6H_2_O (5)

Ammonium magnesium phosphate is a relatively stable corrosion product, and thus, it is a useful component in the corrosion film to protect the AZ91D magnesium alloy against corrosion. In fact, it is the main component in the micro-arc oxidation film and chemical conversion film in previous studies [21,22]. A low concentration of phosphate is favorable for the corrosion of the AZ91D magnesium alloy. Furthermore, corrosion is accelerated when the metabolites of SRB inevitably interact with the corrosion film. Both the magnesium ions and the phosphate ions are necessary for the metabolic activities. Magnesium is an important component of the activated agent to the enzyme, and phosphate is an important component of the cell membrane. Therefore, SRB directly influence the reaction mentioned in Formula (5). These vital components are obtained by the degradation of minerals. However, in the corrosion experiments, these substances may be obtained from the corrosion products. Therefore, this could remarkably influence the corrosion behavior of the AZ91D magnesium alloy. Maybe, this is the reason why SRB could accelerate the nucleation and growth of the corrosion pits on the surface of the AZ91D magnesium alloy.

For the AZ91D magnesium alloy, cracks easily occur at the α phase/β phase interface, due to the different deformability of the two phases [23]. In this case, stress concentration emerges at the α phase/β phase interface, and therefore, the micro cracks easily nucleate in the interface.

In the presence of SRB, pitting corrosion formed on the surface of the AZ91D magnesium alloy. Corrosion pits could remarkably change the stress distribution and cause the stress concentration at the bottom of corrosion pits. Some secondary cracks in the corrosion pit confirmed the occurrence of the stress concentration. In a word, the SRB accelerate the drop in the corrosion residual strength by their microbiological activity.

## 3. Experimental Section

### 3.1. Sample Preparation

A commercial cast AZ91D magnesium alloy was adopted, and its composition is analyzed by inductively coupled plasma atomic emission spectrometry (ICP-AES, 8000I, China) and the result is listed in Table 1. Tensile samples were cut from the cast ingots and the lathed consulted ASTM B557 standard [24]. Subsequently, all samples were polished until 1200 grit SiC papers, washed using absolute ethyl alcohol and dried in cold flowing air before the corrosion experiments. Samples were wrapped in the filter paper and stored in desiccators.

**Table 1:** Chemical composition of the AZ91D alloy measured by ICP-AES.

AZ91D alloy element	Al	Zn	Mn	Si	Fe	Cu	Ni	Mg
**Element composition, wt%**	7.98	0.60	0.26	0.02	0.001	0.002	0.001	–

### 3.2. Cultivation of SRB

The sulfate-reducing bacteria (SRB) used in the experiments were isolated from marine environments and cultivated in American petroleum institute (API) recommended liquid culture medium. The compositions of the culture media and their concentrations are given in Table 2. The pH value of the liquid culture media was adjusted to 7.25 ± 0.05. The chemicals, ascorbic acid (0.1 g/L) and ammonium ferrous sulfate (0.05 g/L), which were sterilized by ultraviolet radiation for 30 min, were added into the triangular flask after autoclaving. All vessels used in the experiment were sterilized by autoclaving. SRB should be stored at −4 °C before the experiments. During the experiment, the SRB were inoculated into the liquid culture media. Subsequently, 30 mL liquid paraffin was added into the culture containers in order to stop the O_2_ from diffusing into the culture media and creating an anaerobic environment. The SRB were cultivated in a constant temperature incubator at 37 °C until the whole triangular flask turned black. In the following corrosion experiments, the SRB were applied.

**Table 2:** Main composition of the culture media and their concentrations.

Medicine	Purity	Concentration
Sodium sulfate	Analytical reagent grade (≥99.5%)	0.5 g/L
Ammonium chloride		1.0 g/L
Calcium chloride		0.1 g/L
Ammonium chloride		1.0 g/L
Di-potassium hydrogen orthophosphate		0.5 g/L
Magnesium sulfate		2.0 g/L
Sodium lactate		3.5 g/L
Yeast extract		1.0 g/L

### 3.3. Corrosion Experiments

All of the corrosion experiments were carried out in a sterile chamber. The SRB strain was inoculated into the sterilized culture media, and simultaneously, the test samples were immersed into the culture media with and without SRB for 12, 60, 240, 372 and 430 h in order to provide some comparative results. At each time point, three samples were taken out from the corrosion media, washed with absolute ethyl alcohol and dried in cold flowing air. Then, the test samples were put into the valve bags, which were numbered and marked beforehand. In the corrosion experiments, the pH value of the corrosion media was tested by a 3C digital pH meter.

### 3.4. Morphology of the Corrosion Product and Fractography

The morphology of the corrosion product and fractography were recorded by a JSM-5600 scanning electron microscopy (SEM, JEOL, Tokyo, Japan) at an accelerating voltage of 20 kV. A camera was adopted to record the surface state of samples.

### 3.5. Tensile Experiments

Tensile tests were carried out on a materials test machine (AG-10TA, Shimadzu, Kyoto, Japan) with a strain rate of 5.56 × 10^−4^ s^−1^. In order to ensure the accuracy of experiment result, at least three samples for each corrosion time were tested, and the average value was calculated as the experiment result.

## 4. Conclusions

The purpose of this paper was to investigate the effect of SRB on the corrosion residual strength of the AZ91D magnesium alloy. Analysis of the morphology of the corrosion product, the composition of the corrosion film and the fracture provide some useful information to understand the corrosion residual strength of the AZ91D magnesium alloy influenced by SRB. Conclusions can be drawn as follows:

(1) The phosphate element in the corrosion film of the AZ91D magnesium alloy decreased in the presence of SRB;
(2) The metabolism of SRB changes the local corrosion environment and leads to an accelerated pitting corrosion;
(3) The corrosion residual strength of the AZ91D magnesium alloy drops quickly in the SRB corrosion media due to the rapid pitting corrosion induced by SRB.

## References

[1] Amira S., Huot J. (2012). Effect of cold rolling on hydrogen sorption properties of die-cast and as-cast magnesium alloys. J. Alloys Compd..

[2] Sivapragash M., Lakshminarayanan P.R., Karthikeyan R., Hanumantha M., Bhatt R.R. (2008). Hotdeformation behavior of ZE41A magnesium alloy. Mater. Des..

[3] Altun H., Sen S. (2006). The effect of PVD coatings on the corrosion behaviour of AZ91 magnesium alloy. Mater. Des..

[4] Altun H., Sinici H. (2008). Corrosion behaviour of magnesium alloys coated with TiN by cathodic arc deposition in NaCl and Na_2_SO_4_ solutions. Mater. Charact..

[5] Walter R., Bobby M. (2011). Influence of surface roughness on the corrosion behaviour of magnesium alloy. Mater. Des..

[6] Zhang S.Y., Lia Q., Yang X.K., Zhong X.K., Dai Y. (2010). Corrosion resistance of AZ91D magnesium alloy with electroless plating pretreatment and Ni-TiO_2_ composite coating. Mater. Charact..

[7] Aghayani M.K., Niroumand B. (2011). Effects of ultrasonic treatment on microstructure and tensile strength of AZ91 magnesium alloy. J. Alloys Compd..

[8] Barrena M.I., Gómez de Salazar J.M., Matesanz L., Soria A. (2011). Effect of heat treatments on oxidation kinetics in AZ91 and AM60 magnesium alloys. Mater. Charact..

[9] Wang L., Zhang B.P., Shinohara T. (2010). Corrosion behavior of AZ91 magnesium alloy in dilute NaCl solutions. Mater. Des..

[10] Jönsson M., Persson D., Leygraf C. (2008). Atmospheric corrosion of field-exposed magnesium alloy AZ91D. Corros. Sci..

[11] Zhou W., Shen T., Aung N.N. (2010). Effect of heat treatment on corrosion behaviour of magnesium alloy AZ91D in simulated body fluid. Corros. Sci..

[12] Zhang T., Li Y., Wang F.H. (2006). Roles of β phase in the corrosion procss of AZ91D magnesium alloy. Corros. Sci..

[13] Song D., Ma A.B., Jiang J.H., Lin P.H., Yang D.H. (2011). Corrosion behavior of bulk ultra-fine-grained AZ91D magnesium alloy fabricated by equal-channel-angular-pressed. Corros. Sci..

[14] Hu R.G., Zhang S., Bu J.F., Lin C.J., Song G.L. (2012). Recent progress in corrosion of magnesium alloys by organic coatings. Prog. Org. Coat..

[15] Liu Y.H., Wang Q., Song Y.L., Zhang D.W., Yu S.R., Zhu X.Y. (2009). A study on the corrosion behavior of Ce-modified cast AZ91 magnesium alloy in the presence of sulfate-reducing bacteria. J. Alloys Compd..

[16] Li C.F., Liu Y.H., Wang Q., Zhang L.N., Zhang D.W. (2010). Study on the corrosion residual strength of the 1.0 wt% Ce modified AZ91 magnesium alloy. Mater. Charact..

[17] Wang Q., Liu Y.H., Zhu X.Y., Yu S.R., Zhang L.N. (2009). Study on the effect of corrosion on the tensile properties of the 1.0 wt% Yttrium modified AZ91 magnesium alloy. Mater. Sci. Eng. A.

[18] Gang C., Peng J., Park K. (1994). Electrochemical mechanisms of anaerobic corrosion influenced by sulfate-reducing bacteria. Water Res..

[19] Duan J.Z., Hou B.R., Yu Z.G. (2006). Characteristics of sulfide corrosion products on 316L stainless steel surfaces in the presence of sulfate-reducing bacteria. Mater. Sci. Eng. C.

[20] Fang S.J., Liu Y.H., Qiao J., Zhang W. (2011). Influence of SRB on corrosion behaviour of AZ91 magnesium alloy in two kinds of culture media. J. Mater. Eng..

[21] Forno A.D., Bestetti M. (2010). Effect of the electrolytic solution composition on the performance of micro-arc anodic oxidation films formed on AM60B magnesium alloy. Surf. Coat. Technol..

[22] Gu Y.H., Bandopadhyay S., Chen C.F., Guo Y.J., Ning C.Y. (2012). Effect of oxidation time on the corrosion behavior of micro-arc oxidation produced AZ31 magnesium alloys in simulated body fluid. J. Alloys Compd..

[23] Lü Z., Wang Q.D., Ding W.J., Zeng X.Q., Zhu Y.P. (2000). Fracture behavior of AZ91 magnesium alloy. Mater. Lett..

[24] ASTM International (2010). Standard Test Methods for Tension Testing Wrought and Cast Aluminum-and Magnesium-Alloy Products.

